# A randomized controlled trial of the influence of yoga for women with symptoms of post-traumatic stress disorder

**DOI:** 10.1186/s12967-022-03356-0

**Published:** 2022-04-05

**Authors:** Lei Yi, Yunling Lian, Ning Ma, Ni Duan

**Affiliations:** 1 The Third Department, Qingdao Mental Health Center, No. 299 Nan Jing Road, Qingdao, 266034 Shandong China; 2 Department of Geriatrics, Qingdao Mental Health Center, No. 299 Nan Jing Road, Qingdao, 266034 Shandong China

**Keywords:** Posttraumatic stress disorder, Motor vehicle accident, Yoga, Depression, Anxiety

## Abstract

**Background:**

Survivors in motor vehicle accident (MVA) may have posttraumatic stress disorder (PTSD). Yoga is a complementary approach for PTSD therapy.

**Methods:**

This randomized controlled trial explored whether yoga intervention has effects on reducing the symptoms of PTSD in women survived in MVA. Participants (n = 94) were recruited and randomized into control group or yoga group. Participants attended 6 45-minuite yoga sessions in 12 weeks. Depression Anxiety Stress Scales (DASS) and Impact of Events Scale-Revised (IES-R) were used to assess psychological distress.

**Results:**

Post-intervention IES-R total score of yoga group was significantly lower than that of control group (p = 0.01). At both post-intervention and 3-months post intervention, the DASS-21 total scores of yoga group were both significantly lower than those of control group (p = 0.043, p = 0.024). Yoga group showed lower anxiety and depression level compared to control group at both post-intervention (p = 0.033, p < 0.001) and post-follow-up (p = 0.004, p = 0.035). Yoga group had lower levels of intrusion and avoidance compared to control group after intervention (p = 0.002, p < 0.001).

**Conclusion:**

Results illustrate that yoga intervention may alleviate anxiety and depression and improve the symptoms of PTSD in women with PTSD following MVA.

## Introduction

Having the experience of motor vehicle accident (MVA) will trigger the generation of psychosocial impacts and psychological disorders [1]. The psychosocial impacts caused by MVA include disability, chronic pain, trauma, and loss of income [2]. Post-traumatic stress disorder (PTSD) and major depressive disorder (MDD) and are two major psychological disorders in MVA [3]. Research has reported that in MVA survivors, 21–67% of them suffer depressive, 47% have driving phobia, and 20–40% suffer PTSD [4]. Up to at least 12 months post-MVA, the incidence of MDD and PTSD remain elevated [5]. A systematic review has demonstrated that in people sustaining MVA, the median occurrence of PTSD was 30% after 1 month and had a declining trend to 15% after 12 months [6]. Another study illustrated that the rate of MVA survivors with probable PTSD was around 30% within 4 weeks and 20% after 6 months [7]. PTSD are associated with a dysregulation of both neuroendocrine system and renin angiotensin system [8].

To reduce the risk of psychological distress caused by MVA, interventions are performed and proved to be effective. Cognitive behavior therapy which enhances the adaptive psychological, social, and behavioral skills of survivors is effective in the improvement of psychological symptoms after MVA [9].

As a non-pharmacologic strategy, yoga combines the practice of meditation, physical postures, and breathing [10]. Recently, yoga has been utilized to reduce stress and improve psychological disorder [11]. In a number of populations, yoga is effective in reducing the PTSD symptoms [12]. It has been proved that in PTSD patients, the performance of yoga reduces PTSD-caused physiological arousal and inhibits PTSD pathology through improving somatic regulation and body awareness [13].

Since yoga is able to ameliorate psychological and physical conditions of PTSD patients, this randomized controlled trial aimed to evaluate the effect of yoga on reducing symptoms of PTSD in women after MVA.

## Methods

### Participants

This study was approved by the Ethics Committee of Qingdao Mental Health Center. Eligible participants were adult women diagnosed with MVA-related PTSD that happened at least 3 months ago. PTSD was diagnosed by meeting Diagnostic and Statistical Manual of Mental Disorders 4th Edition (DSM-IV) PTSD criterion A1 for a traumatic event. The exclusion criteria were: (1) Having organic mental disorder, symptomatic bipolar disorder, psychotic disorder, or schizophrenia; (2) Having brain surgery history, brain damage, or neurological problems; (3) Having substance abuse or currently suicidal.

### Procedures

**Fig. 1:**
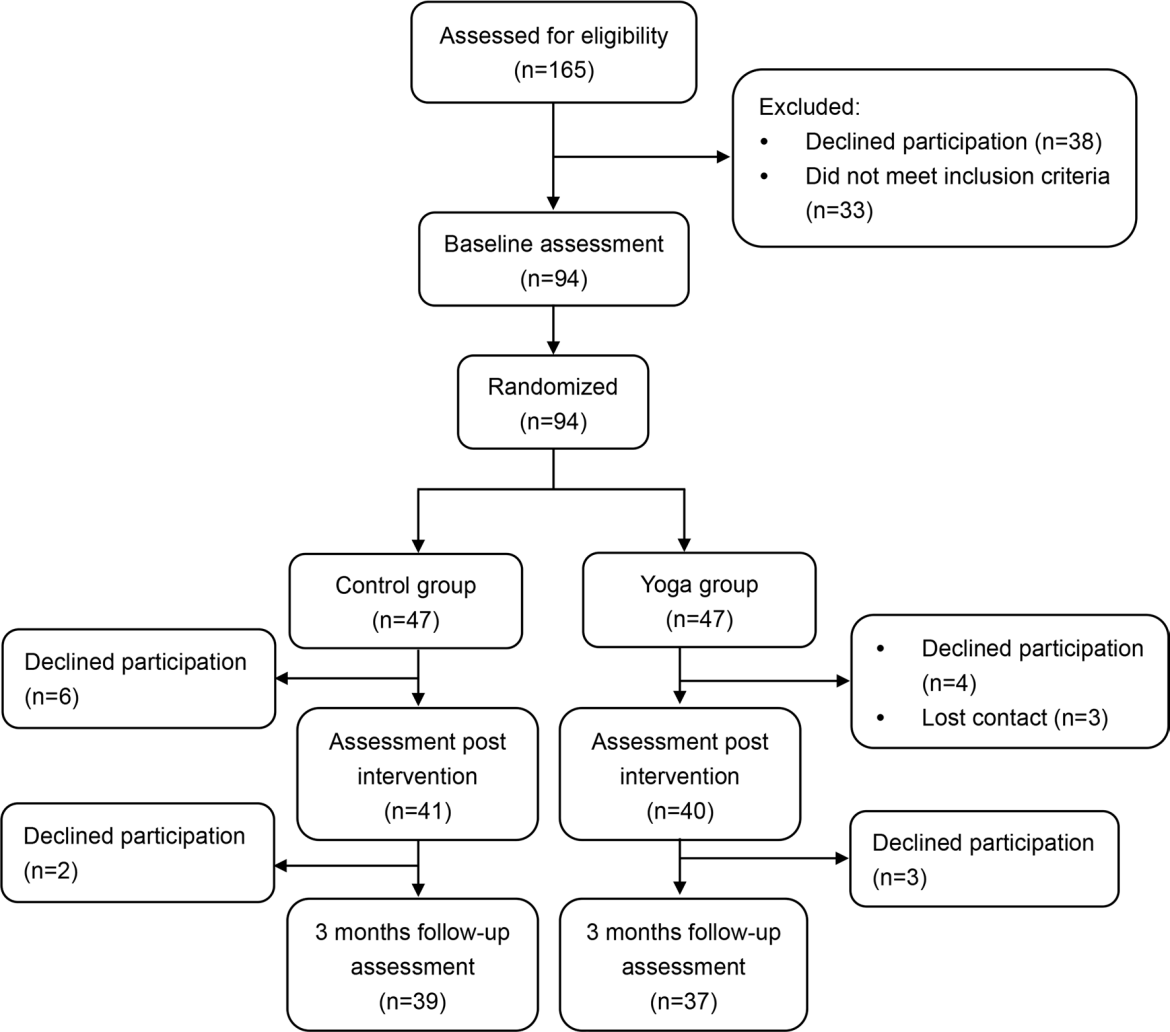
CONSORT flow diagram

Figure 1 showed the flow for recruitment, intervention, and assessment processes. A total of 165 individuals were assessed for eligibility, of whom 94 were included in this trail. After completing baseline measures, these 94 patients were randomly divided into yoga group (n = 47) and control group (n = 47) through a web-based randomization system. Participants in yoga group attended 6 45-minuite yoga sessions in 12 weeks. In the control group, yoga sessions were replaced by exchanging daily life experiences and playing board games in 12 weeks. After intervention and 3-month follow-up, participants in both groups were assessed. For the 47 participants in yoga group, 3 lost contact, 7 withdrew, and 37 completed. 39 participants in control group completed this trail after 8 participants withdrew.

### Intervention

Participants in yoga group were provided 12-week yoga sessions developed by the research team, 1 session in every 2 weeks. Yoga sessions were held in a specific meeting room and performed under the instruction of a yoga instructor. The style of yoga in this trail was Kripalu. This kind of yoga focuses on compassionate self‐observation, building connections between mind and body on the present moment. The yoga sessions were designed to adapt for all body types and be noncompetitive. The contents of yoga sessions were shown in Table 1.

**Table 1:** Contents of yoga sessions

	Time (min)	Content
Lesson 1	45	Notice the breath coming into your body and leaving your body. Notice how you feel
Lesson 2	45	Focus on how you feel in a yoga pose: how do your legs and feet feel? What do your arms and hands feel? What emotions are you experiencing?
Lesson 3	45	Pay attention to what the body feels in a yoga pose instead of how you look
Lesson 4	45	Challenge yourself to stay in a pose longer, even if it is slightly uncomfortable, and practice to tolerate the discomfort. Learn to notice the emotions and accept situations for the way they are
Lesson 5	45	Relax yourself and release your stress. Create the experience that allows you to let go
Lesson 6	45	Image putting bad emotions aside when you start your yoga practice

### Assessments

In this trail, psychometric assessments included the Impact of Events Scale-Revised (IES-R) and the Depression, Anxiety and Stress Scale (DASS-21). All the measures were collected at baseline, post-intervention, and 3-month follow-up.

IES-R was employed to evaluate PTSD symptoms. This self-report measure consists of 22 items. The score of each item is from 0 to 4 (0 = not at all, 1 = a little bit, 2 = moderately, 3 = quite a bit, 4 = extremely). There are three subscales, avoidance, intrusion, and hyperarousal. The total score is the sum of the three subscales scores, ranges from 0 to 88. Higher IES-R score indicates worse symptom.

DASS-21 was employed to assess the severity of psychological distress. This self-report scale has 21 items to evaluate anxiety, depression, and stress. The score of each item is from 0 to 3 and the sum of the scores of items is total score. Higher score shows stronger symptom of anxiety, depression, and stress.

### Data analysis

In this research, statistical analyses were performed by SPSS version 17.0 software. Data were presented as mean ± standard deviation (SD) or proportion (%). Differences in continuous variables between different groups were compared by ANOVA test. *p < 0.05, **p < 0.01, ***p < 0.001, ns: no significance.

## Results

### Demographics

Table 2 showed the participants’ characteristics in two groups. Based on the result of statistical analysis, no significant differences were found between the participants in two groups on any of the characteristics, such as age, body mass index (BMI), education level, marital status, employment status, role in MVA, injury severity, days since MVA, or perceived life threat to self (all p > 0.05).

**Table 2:** Comparison of participant characteristics between yoga and control groups

	Yoga group (n = 37)	Control group (n = 39)	p value
Age (years)	40.8 (13.2)	42.1 (15.9)	0.699
BMI (kg/m^2^)	28.4 (5.0)	27.2 (4.4)	0.271
Education			
High school or lower	20 (54.1)	28 (71.8)	0.109
University or higher	17 (45.9)	11 (28.2)	
Marital status			
Single	8 (21.6)	6 (15.4)	0.422
Married/in a relationship	23 (62.2)	22 (56.4)	
Divorced/widowed	6 (16.2)	11 (28.2)	
Employment status			
Full time	18 (48.6)	21 (53.8)	0.576
Part time	8 (21.6)	6 (15.4)	
Unemployed	6 (16.2)	8 (20.5)	
Retired	3 (8.1)	4 (10.3)	
Student	2 (5.4)	0 (0.0)	
Role in MVA			
Driver	21 (56.8)	19 (48.7)	0.884
Passenger	7 (18.9)	9 (23.1)	
Motorbike rider	5 (13.5)	7 (17.9)	
Cyclist	3 (8.1)	2 (5.1)	
Pedestrian	1 (2.7)	2 (5.1)	
Injury severity	1.37(1.02)	1.30 (0.95)	0.758
Days since MVA	56.3 (18.4)	63.8 (20.1)	0.094
Perceived life threat to self	3.07 (1.10)	3.25 (1.04)	0.466

*BMI* body mass index, *MVA* motor vehicle accident

Data are shown as mean (SD) or n (%)

### Total scores of IES-R and DASS-21

**Fig. 2:**
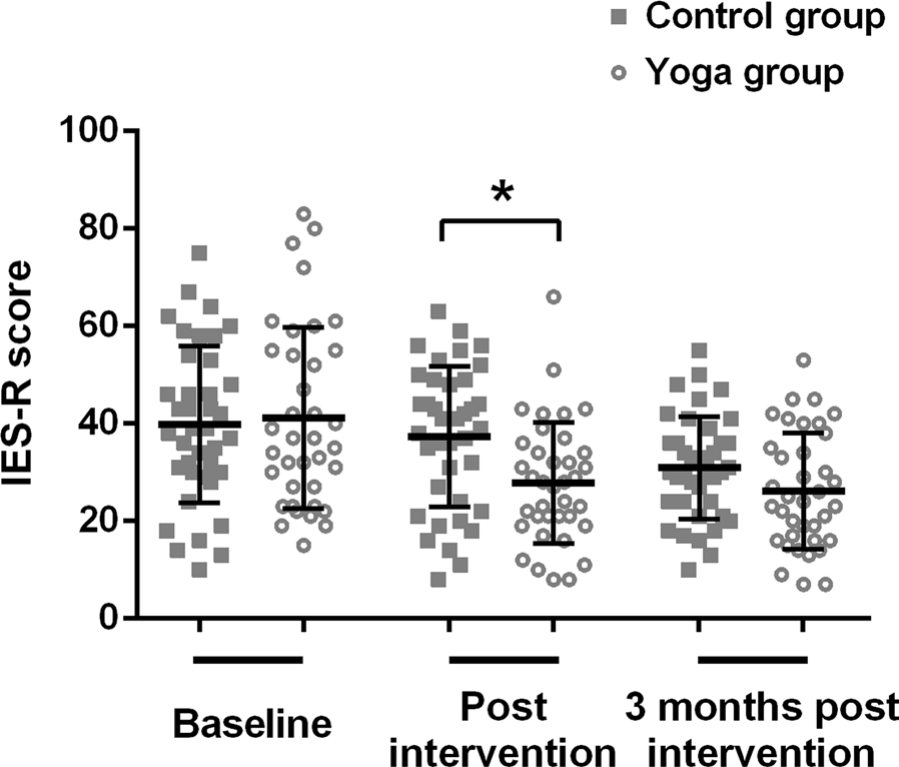
IES-R total scores for the two groups by time point. Data were shown as mean ± SD. *p < 0.05 compared with control group. *IES-R* impact of event scale-revised

IES-R total scores of participants in these two groups were shown in Fig. 2. In both groups, IES-R total scores decreased throughout the trial. No significant differences were found between two groups on IES-R total scores at baseline and post-follow-up (all p > 0.05). However, post-intervention IES-R total score of yoga group was significantly lower than that of control group (p = 0.01).

**Fig. 3:**
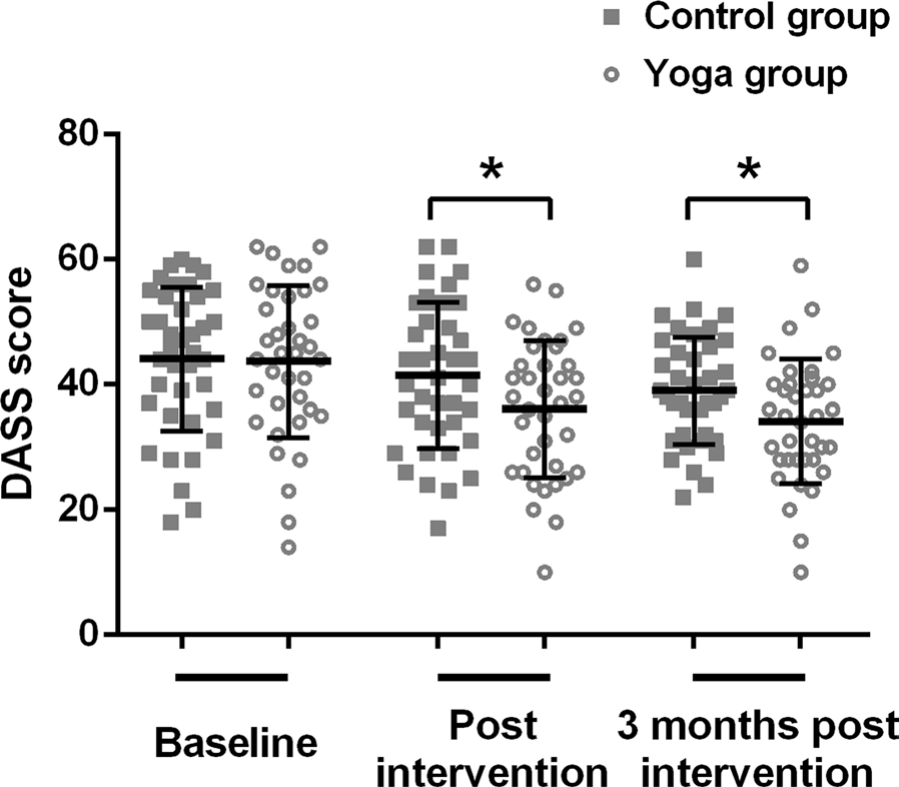
DASS total scores for the two groups by time point. Data were shown as mean ± SD. *p < 0.05 compared with control group. *DASS* depression, anxiety, and stress scale

DASS-21 total scores were shown in Fig. 3. DASS-21 total scores showed reductions over time for both groups. At baseline, the DASS-21 total scores in both groups had no significant difference (p > 0.05). At both post-intervention and 3-months post intervention, the DASS-21 total scores of yoga group were both significantly lower than those of control group (p = 0.043, p = 0.024).

### The scores of subscales in DASS-21 and IES-R

The scores of subscales in DASS-21 and IES-R were presented in Table 3. No significant differences were found between groups for baseline IES-R and DASS-21 subscales. For DASS-21, however, the level of stress was not influenced by the intervention of yoga. For IES-R, participants in yoga group had lower levels of intrusion and avoidance compared to control group after intervention (p = 0.002, p < 0.001). At 3-months post intervention, intrusion level and avoidance level in the yoga group were significantly lower than in the control group (p = 0.037).

**Table 3:** IES-R and DASS for control and yoga groups at baseline and post intervention

		Yoga group (n = 37)	Control group (n = 39)	p value
DASS				
Baseline	Depression	14.3 (5.1)	14.8 (4.7)	0.659
	Anxiety	12.2 (3.5)	13.3 (4.1)	0.212
	Stress	20.0 (8.6)	19.1 (7.7)	0.633
	Total	46.5 (15.6)	47.2 (16.2)	0.848
Post intervention	Depression	11.6 (3.4)	13.5 (4.2)	0.033
	Anxiety	9.1 (2.9)	11.9 (3.3)	< 0.001
	Stress	15.4 (6.4)	16.7 (7.8)	0.429
	Total	36.1 (11.2)	42.1 (14.1)	0.043
3 months post intervention	Depression	10.5 (2.9)	12.8 (3.8)	0.004
	Anxiety	8.8 (3.0)	10.3 (3.1)	0.035
	Stress	14.8 (5.2)	15.9 (4.6)	0.333
	Total	34.1 (9.9)	39.0 (8.5)	0.024
IES-R				
Baseline	Intrusion	16.3 (7.2)	15.6 (7.7)	0.683
	Avoidance	15.1 (6.5)	13.8 (5.9)	0.365
	Hyperarousal	10.1 (5.7)	9.8 (4.8)	0.805
	Total	41.5 (19.5)	39.2 (17.2)	0.588
Post intervention	Intrusion	10.4 (5.1)	14.5 (6.0)	0.002
	Avoidance	9.2 (3.9)	12.6 (4.4)	< 0.001
	Hyperarousal	8.1 (3.2)	9.4 (4.8)	0.167
	Total	27.7 (12.8)	36.5 (16.1)	0.010
3 months post intervention	Intrusion	9.4 (4.2)	11.5 (4.4)	0.037
	Avoidance	9.2 (3.9)	10.9 (3.5)	0.005
	Hyperarousal	7.6 (3.1)	8.6 (2.8)	0.145
	Total	26.2 (12.0)	31.0 (10.5)	0.068

DASS, Depression, anxiety, and stress scale; IES-R, Impact of Event Scale – Revised

Data are shown as mean (SD)

## Discussion

This randomized controlled trail examined a 12-week yoga intervention’s effect on psychological distress in women with PTSD following MVA. The preliminary results demonstrated that both yoga group and control group showed trends for the improvement of psychological distress, while the intervention of yoga promoted the recovery of psychological disorders in women with PTSD following MVA. There were significant reductions in depression, anxiety, and intrusion in yoga group as compared to control group after 12-week intervention and 3-month follow-up. Additionally, the intervention of yoga had no notable influence on stress and hyperarousal in women with PTSD following MVA.

PTSD refers to a series of clinical symptoms shown by the patient's psychological and physical excessive stress response after personally experiencing a serious traumatic event that causes or may lead to death or physical injury [14]. With the deepening of research, PTSD is widely present in people who have experienced all serious traumas including violent events and natural disasters [15]. In modern society, more attention has been paid to the psychological disorder caused by traffic accidents, including PTSD. It has been reported that the incidence of MVA-caused PTSD ranges from 8.5% to 23.1% [16]. However, in China, the patients' physiological problems caused by traffic accidents have been effectively treated, while the psychological problems such as PTSD have not received enough attention and corresponding treatment, which often increases the patient's suffering and prolongs the treatment. PTSD has association with both dysregulation of neuroendocrine system and renin–angiotensin–aldosterone-system. Traumatization has lasting and cumulative effects on the activity of renin–angiotensin–aldosterone-system and elevated renin levels may increase the risk for developing PTSD and other disease [8, 17].

In recent years, yoga has been considered as complementary and alternative medicine [18]. Yoga is a combination of breathing techniques, meditation, physical postures, and relaxation which have benefits for both physical and mental conditions. Yoga in the school setting is a viable and potentially efficacious strategy for improving child and adolescent health [19].

Meditation and yoga are promising complementary approaches in the treatment of PTSD among adults [20]. A study has demonstrated the preliminary efficacy and feasibility of online yoga on alleviating symptoms of PTSD, anxiety, and depression in women who have experienced stillbirth [10]. A randomized controlled trial in women with PTSD has proved that Kripalu-based yoga intervention reduces expressive suppression and improves PTSD symptoms through increasing psychological flexibility [21]. Another research also showed that a 12-session Kripalu-based yoga intervention plays a role in the alleviation of reexperiencing and hyperarousal symptoms in women with PTSD [21]. Yoga may be an effective alternative to trauma-focused therapy for women veterans with PTSD [22]. Researchers have illustrated that yoga practice has association with decreased sympathetic activity, increased parasympathetic activity, and down-regulated hypothalamic–pituitary–adrenal axis and positive effects on cognitive activity [23, 24]. Practicing yoga triggers the adjustment of cognition and behavior through enhancing mind–body awareness, increases behavioral activation in pleasant activities, improves emotion‐regulation skill, and reduces reexperiencing and avoidance symptoms [25–27]. Yoga has positively impact on PTSD symptoms and may be an effective adjunctive treatment for PTSD. Thus, in this research, we explored whether Kripalu-based yoga intervention contributed to the improvement of psychological distress in women with PTSD following MVA.

In this trail, participants in yoga group were provided 12-week Kripalu-based yoga sessions. Sessions were held once every 2 weeks. Kripalu-based yoga emphasizes the connections between mind and body. Kripalu yoga is often described as "dynamic meditation", focusing less on the physical details of the yoga postures and more on the emotional and psychological feelings they bring to the person, thus requiring the student to maintain a gentle, compassionate and introspective attitude. This kind of yoga believes that the body has its own wisdom and sends out messages to prompt the individual to practice the yoga postures in a fluid and natural way. Each yoga pose is held for a long period of time in order to uncover or release repressed emotions. Kripalu yoga may have potential as a PTSD therapy in a veteran or military population [28].

In 47 participants of yoga group, only 6 dropped out during 12-week intervention. Thus, most of participants were satisfied with the intervention. Time, mood, and stress might be the major reason for not completing the study. When examined separately for the two groups, the IES-R total scores and DASS-21 total scores both decreased for both groups after 12-week intervention and 3-month follow-up. IES-R total scores in yoga group was significantly lower than in control group postintervention. DASS-21 total scores also had significant differences between groups postintervention and post-follow-up. Thus, the 12-week intervention of yoga enhanced the improvement of psychological distress in women with PTSD following MVA. Decreased scores of IES-R and DASS-21 indicated that clinical improvement in the PTSD symptoms of women also happened in control group. It was possible that the exchanging of daily life experiences and playing board games enhanced the communication between patients and contributed to the improvement of psychological distress. Alternatively, other unmeasured variables may also account for the improvement in control group. When compared with control group, there were significant reduction in anxiety and depression symptoms at post-intervention and follow-up, but not in stress. The intervention of yoga might have no significant effect on alleviating stress caused by PTSD in woman survived from MVA. Results also demonstrated the short-term effects of yoga on intrusion and avoidance symptoms and the long-term effect on intrusion in women with PTSD following MVA. These findings provided preliminary evidence to suggest that yoga may reduce psychological distress and improve PTSD symptoms among women with PTSD following MVA.

Several limitations of the research should be noted. First, the limitation of time and funding caused a relatively small sample size. Limited sample size might influence the detection of differences both between and within groups and limit the generalizability of conclusion. Second, we only evaluated the influence of the Kripalu-based yoga intervention on the participants. Since many different styles of yoga exist, different types might have diverse effects on physical and mental health and PTSD symptom reduction. Kripalu-based yoga might not be the most beneficial type. Although Kripalu-based yoga reduced psychological distress and improve PTSD symptoms, which specific aspect of the yoga practice in this trail was most effective was not evaluated. Third, in this trail, we utilized self-report data. Data might be influenced by the expectancy effects of patients and the results were subject to biases that are inevitable with this type of data.

## Conclusion

In conclusion, the intervention of a 12-week yoga practice may be an effective and feasible strategy to reduce psychological distress in women with PTSD following MVA. The results of this study suggest that alleviating PTSD after a car accident through yoga is very feasible and can be sustained in daily life. PTSD from car accidents remains a noteworthy social problem that requires effective and feasible programs to alleviate PTSD in order to prevent negative health effects. The results of this study support the use of Kripalu yoga to improve the mental health of women with PTSD who have experienced a major car accident.

## Data Availability

All data generated or analyzed during this study are included in this published article.
